# Exploration of social determinants of health and prostate cancer prevalence in the US: a cross-sectional study of NHANES data from 2003 to 2010

**DOI:** 10.3389/fpubh.2025.1564498

**Published:** 2025-03-12

**Authors:** Danfei Hu, Xiaodong Chen, Mingyao Li, Huacai Xiong, Xuefeng Lu, Feng Chen

**Affiliations:** ^1^Department of Radiation Oncology, Ningbo Medical Center Lihuili Hospital, Ningbo, China; ^2^Department of Urology, Ningbo Urology and Nephrology Hospital, Ningbo, China

**Keywords:** social determinants of health, prostate cancer, prostate-specific antigen, prevalence, NHANES

## Abstract

**Objective:**

Social determinants of health (SDoH) are increasingly recognized as key factors in addressing health inequities. This study aimed to explore the association between SDoH and risk of prostate cancer (PCa).

**Methods:**

We analyzed data from the National Health and Nutrition Examination Survey 2003–2010. PCa diagnosis was based on self-reported questionnaires, while highly-probable PCa was assessed using prostate-specific antigen levels. Multivariate logistic regression, restricted cubic spline, and subgroup analysis were performed. Three models were employed: the crude model (unadjusted), model 1 (adjusted for age and race/ethnicity), and model 2 (further adjusted for body mass index, alcohol consumption, and smoking status).

**Results:**

The median age of 5,633 participants was 54 years. A negative association was found between the SDoH score and PCa prevalence (OR = 0.868, 95% CI: 0.786–0.959, *p* = 0.006). Specifically, a family income-to-poverty ratio < 3 (OR = 0.69, 95% CI: 0.499–0.954, *p* = 0.029) and lack of healthcare access or reliance on emergency rooms (OR = 0.429, 95% CI: 0.218–0.842, *p* = 0.017) were independently associated with lower PCa prevalence. In model 2, no significant association was found between SDoH and highly probable PCa. A linear association between SDoH and PCa prevalence was observed. A consistently negative association was noted among participants aged ≥ 60 years, Non-Hispanic Black, Non-Hispanic White, and non-obese individuals.

**Conclusions:**

The negative association between SDoH and PCa prevalence is likely attributable to inadequate screening and underreporting, rather than any protective effects. Unfavorable SDoH is not a risk factor for the onset of PCa. This study underscores the importance of addressing disparities in healthcare access and improving equity in PCa screening.

## 1 Introduction

The global incidence of prostate cancer (PCa) is projected to increase from 1.4 million cases annually in 2020 to 2.9 million by 2040, with annual deaths expected to rise from 375,000 to nearly 700,000 (1). PCa is the most diagnosed cancer and the second leading cause of cancer deaths in American men, with one of the largest racial disparities in outcomes in oncology (2). Black men are disproportionately affected by PCa, presenting at an earlier stage, with more aggressive disease, and experiencing higher mortality rates compared with White men (3, 4). Despite substantial research investment to identify biological causes for these disparities, evidence supporting specific biological factors remains limited (5).

As the field of medicine strives for equity in care, research that highlights the association between social determinants of health (SDoH) and poorer healthcare outcomes is essential to inform quality improvement strategies. The SDoH framework provides a comprehensive evaluation of how the conditions in which people are born, live, learn, work, play, worship, and age impact health outcomes (6, 7). Emerging evidence suggests that unfavorable SDoH are associated with increased rates of premature death and contribute to differences between Black and White racial groups in premature all-cause mortality in the US population (7). Similarly, the impact of SDoH on outcomes across the cancer prevention and control continuum has widely attracted attention (8, 9). Black men with PCa face persistent disparities in SDoH, resulting in reduced prostate-specific antigen (PSA) screening, limited access to staging imaging, non-guideline-concordant care, a higher burden of comorbidities, and restricted access to curative treatment (3, 10–13). A recent meta-analysis showed that after accounting for established SDoH disparities, Black men with PCa had similar or better survival outcomes compared with White men, highlighting the importance of addressing SDoH to reduce racial disparities in cancer outcomes (14).

However, the association between unfavorable SDoH and PCa prevalence remains unclear. To address this gap, we conducted this study to evaluate the relationship between SDoH and PCa prevalence using cross-sectional data from the National Health and Nutrition Examination Survey (NHANES). We also analyzed the association between SDoH and the risk of high PSA levels, an indicator of highly-probable PCa, in individuals without a PCa diagnosis.

## 2 Methods

### 2.1 Data source and population selection

The NHANES, conducted biennially by the National Center for Health Statistics (NCHS), collects nationally representative data from the non-institutionalized US population using a complex survey design and population-specific sample weights (15). The study protocol was approved by the NCHS Institutional Review Board, and informed consent was obtained from all participants. This study used data from the 2003–2010 NHANES cycles, which included SDoH and PCa questionnaire data. The NHANES dataset is publicly available at https://wwwn.cdc.gov/nchs/nhanes/Default.aspx.

**Figure 1:**
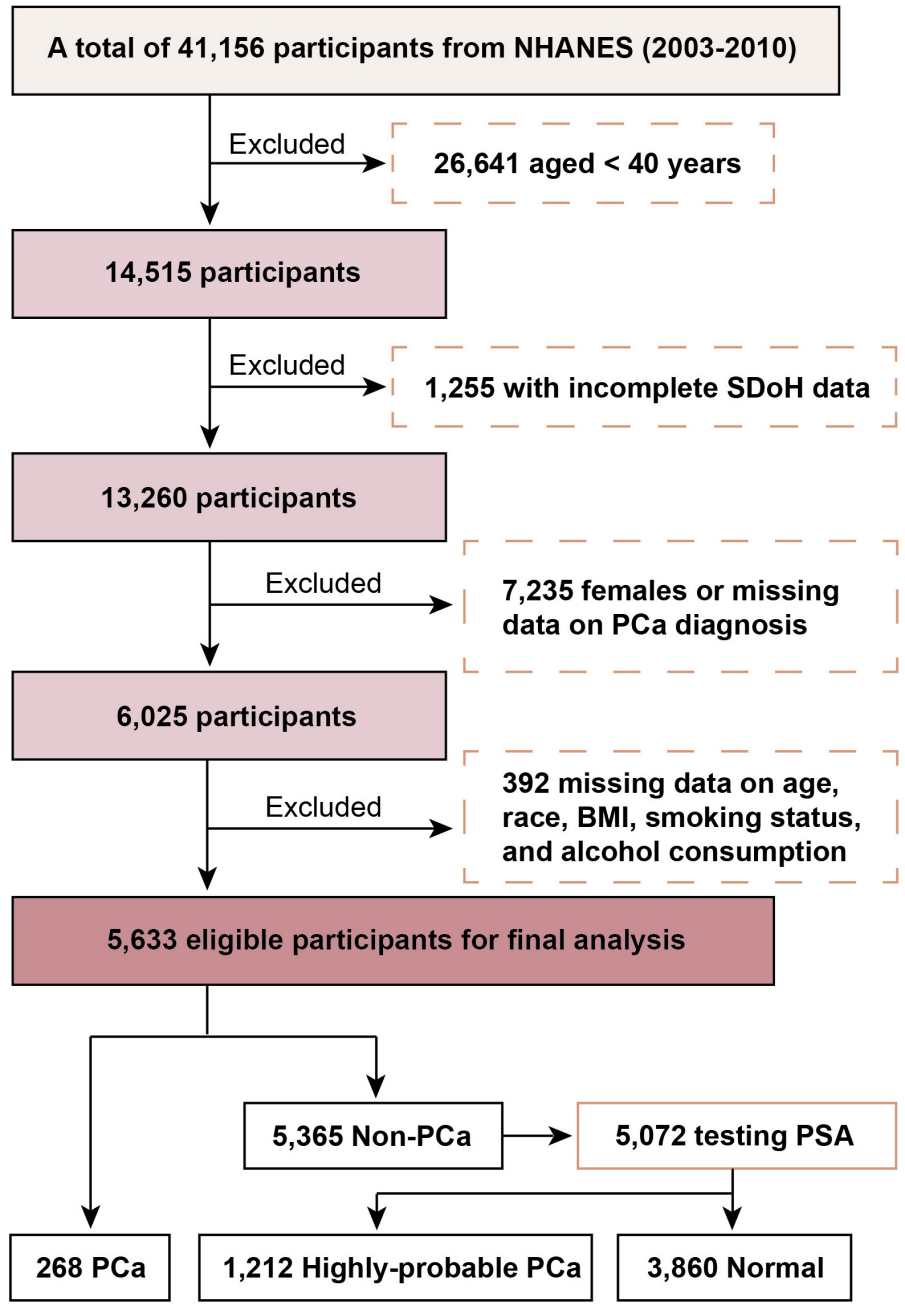
Flowchart of participants selection.

A total of 41,156 NHANES participants were initially considered. Exclusions included: 26,641 individuals under 40 years, 1,255 with incomplete SDoH data, 7,235 females or those missing PCa diagnosis data, and 392 missing data on age, race, BMI, smoking, or alcohol use. The final analytic cohort comprised 5,633 participants (Figure 1).

### 2.2 Assessment of SDoH

The SDoH scoring system was developed based on the definitions outlined in the Healthy People 2030 initiative by the US Department of Health and Human Services (6, 7). This system incorporates variables from five key domains: economic stability, healthcare access and quality, education access and quality, neighborhood and built environment, and social and community context. A total of eight SDoH variables were recorded using standardized NHANES questionnaires: employment status, family income-to-poverty ratio (PIR), food security, education level, healthcare access, health insurance status, homeownership, and marital status. An unfavorable SDoH factor was assigned a score of 1. The total SDoH score ranges from 0 to 8. Detailed descriptions of the SDoH-related questions and definitions are provided in Supplementary Table S1.

### 2.3 Assessment of PCa and highly-probable PCa

Diagnosis of PCa was determined based on questionnaire item KIQ201 in the PSA detection section of the Laboratory Data. Participants were asked, “Have you ever been told by a doctor or health professional that you had PCa?” Those who answered “yes” were identified as having PCa. PSA testing was conducted for male participants aged ≥ 40 years, excluding those with current infection or inflammation of the prostate gland, rectal exam in the past week, prostate biopsy in the past month, cystoscopy in the past month, or a history of PCa. Serum total PSA and free PSA concentrations were measured using immunoassays (Hybritech tests, Beckman Coulter, Fullerton, CA). The PSA ratio was calculated by dividing free PSA by total PSA. Individuals with total PSA levels > 10 ng/mL or between 4 and 10 ng/mL with a PSA ratio < 10% were considered highly-probable PCa (16).

### 2.4 Covariates

Demographic characteristics, such as age and race, were considered confounding factors between SDoH and PCa. Race and ethnicity were classified into four categories: non-Hispanic White, non-Hispanic Black, Mexican American, and other. Data on health-related behaviors were also collected. Smoking status was categorized as never (smoked fewer than 100 cigarettes in their lifetime), former (smoked more than 100 cigarettes in their lifetime but do not currently smoke), and current (smoke ≥ 1 cigarette daily). Alcohol consumption was classified as never (< 12 drinks in lifetime), former (consumed ≥ 12 drinks in a single year or lifetime but did not drink in the past year), and current (≥12 drinks in the past year). Due to a high proportion of missing data on physical activity (up to 44.4%), it was excluded. Body mass index (BMI) was included as a covariate, as it is a well-recognized risk factor for PCa. BMI was classified as underweight (< 18.5 kg/m^2^), normal weight (18.5 kg/m^2^ ≤ BMI < 25 kg/m^2^), overweight (25 kg/m^2^ ≤ BMI < 30 kg/m^2^) and obese (≥30 kg/m^2^).

### 2.5 Statistical analysis

All statistical analyses accounted for the complex survey design and provided population-weighted estimates representative of the non-institutionalized US population from 2003 to 2010. Continuous variables are presented as the weighted means with 95% confidence intervals (CI), while categorical variables are expressed as the percentages (%). Chi-square tests and analysis of variance were utilized to assess differences in baseline characteristics. A multivariate logistic regression model was used to estimate the association of SDoH with PCa, with results presented as odds ratios (OR) and 95% CI. Three models were employed: the crude model (unadjusted), model 1 (adjusted for age and race/ethnicity), and model 2 (further adjusted for BMI, alcohol consumption, and smoking status). A restricted cubic spline (RCS) model was used to graphically represent the dose-response relationship. Subgroup analyses and interaction tests were conducted to explore potential confounding effects. Additionally, we estimated the association of each SDoH with PCa, adjusting for age, race/ethnicity, and other SDoH to identify independent associations. All statistical analyses were performed using R software (version 4.3.3), and a *p* < 0.05 was considered statistically significant.

## 3 Results

### 3.1 Baseline characteristics

Among the 5,633 participants, the median age was 54 years (Table 1). The racial and ethnic composition included 3,112 Non-Hispanic White, 1,060 Non-Hispanic Black, 949 Mexican American, and 512 identifying as other races. Compared to participants without PCa, those with PCa were older and more likely to be Non-Hispanic Black or Non-Hispanic White, former smokers, and former drinkers. Regarding SDoH variables, participants with PCa were more likely to be employed or retired, have a routine place for healthcare, and own their home. The baseline characteristics of 5,072 patients who underwent PSA testing are shown in Supplementary Table S2.

**Table 1:** Characteristics of study participants.

**Variable**	**Total (*n* = 5,633)**	**Non-PCa (*n* = 5,365)**	**PCa (*n* = 268)**	***P*-value**
Age	54.0 (47.0, 65.0)	53.0 (46.0, 64.0)	73.0 (65.0, 79.0)	< 0.0001
**Race/ethnicity**				0.002
Mexican American	949 (6.0)	934 (6.1)	15 (1.8)	
Non-Hispanic Black	1,060 (9.0)	990 (8.9)	70 (11.9)	
Non-Hispanic White	3,112 (77.8)	2,945 (77.7)	167 (81.5)	
Other	512 (7.2)	496 (7.2)	16 (4.8)	
**BMI category**				0.5
Underweight	61 (0.8)	57 (0.8)	4 (0.8)	
Normal	1,282 (21.0)	1,222 (21.0)	60 (22.6)	
Overweight	2,318 (41.9)	2,201 (41.8)	117 (45.1)	
Obese	1,972 (36.3)	1,885 (36.5)	87 (31.5)	
**Alcohol consumption**				0.02
Former	1,503 (21.6)	1,423 (21.4)	80 (29.2)	
Never	371 (5.7)	346 (5.6)	25 (7.7)	
Now	3,759 (72.7)	3,596 (73.0)	163 (63.1)	
**Smoking status**				< 0.0001
Former	2,263 (37.3)	2,123 (36.7)	140 (54.6)	
Never	2,131 (41.2)	2,025 (41.3)	106 (39.2)	
Now	1,239 (21.5)	1,217 (22.0)	22 (6.2)	
**Employment**				< 0.001
Employed, student, retired	4,608 (84.4)	4,365 (84.1)	243 (93.6)	
Not employed	1,025 (15.6)	1,000 (15.9)	25 (6.4)	
**Family income-to-poverty ratio**				0.3
≥3	2,383 (59.2)	2,275 (59.3)	108 (55.4)	
< 3	3,250 (40.8)	3,090 (40.7)	160 (44.6)	
**Food security**				0.1
Full food security	4,505 (86.9)	4,266 (86.7)	239 (92.3)	
Marginal, low, or very low	1,128 (13.1)	1,099 (13.3)	29 (7.7)	
**Education**				0.1
High school or more	3,902 (81.7)	3,719 (81.9)	183 (76.9)	
Less than high school	1,731 (18.3)	1,646 (18.1)	85 (23.1)	
**Access to healthcare**				< 0.0001
Regular health-care facility	4,868 (87.2)	4,608 (86.9)	260 (97.8)	
None or emergency room	765 (12.8)	757 (13.1)	8 (2.2)	
**Health insurance**				0.5
Private insurance	3,192 (68.3)	3,028 (68.4)	164 (65.9)	
Government or none	2,441 (31.7)	2,337 (31.6)	104 (34.1)	
**Housing instability**				0.02
Own home	4,258 (81.5)	4,029 (81.3)	229 (88.3)	
Rent or other arrangement	1,375 (18.5)	1,336 (18.7)	39 (11.7)	
**Marital status**				0.7
Married or living with a partner	4,108 (76.8)	3,908 (76.7)	200 (78.2)	
Not married nor living with a partner	1,525 (23.2)	1,457 (23.3)	68 (21.8)	
**SDoH score**				0.01
0–1	2,268 (55.8)	2,149 (55.7)	119 (57.8)	
2–3	1,796 (26.6)	1,693 (26.4)	103 (31.9)	
4–5	1,164 (13.5)	1,123 (13.6)	41 (9.6)	
≥6	405 (4.2)	400 (4.3)	5 (0.8)	

The data are presented as the mean (95% CI) or number (%).

All estimates were obtained from complex survey designs, using analysis of variance or chi-square tests as appropriate.

PCa, prostate cancer; BMI, body mass index; SDoH, social determinants of health.

### 3.2 Association of SDoH with PCa prevalence

As shown in Table 2, weighted multivariable logistic regression revealed negative associations between both continuous and categorical SDoH scores and the PCa prevalence. After adjusting for age and race/ethnicity (model 1), each unit increase in SDoH score was associated with a 15.6% decrease in PCa prevalence (OR = 0.844, 95% CI: 0.763–0.934, *p* = 0.001). Compared to the lowest SDoH group (0–1), the highest group (≥6) was associated with a lower prevalence of PCa (OR = 0.25, 95% CI: 0.089–0.702, *p* = 0.009), with a significant decreasing trend (*p* for trend < 0.001). In Model 2, each unit increase in SDoH score was associated with a 13.2% decrease in the prevalence of PCa (OR = 0.868, 95% CI: 0.786–0.959, *p* = 0.006). Compared to the lowest SDoH group (0–1), the highest group (≥6) was associated with a decreased prevalence of PCa (OR = 0.313, 95% CI: 0.115–0.854, *p* = 0.024), with a significant decreasing trend (*p* for trend = 0.004).

**Table 2:** Association of SDoH score with PCa prevalence.

**SDoH score**	**Crude model**		**Model 1**		**Model 2**	
	**OR (95% CI)**	***P***	**OR (95% CI)**	***P***	**OR (95% CI)**	***P***
Continuous	0.924 (0.856, 0.997)	0.042	0.844 (0.763, 0.934)	0.001	0.868 (0.786, 0.959)	0.006
**Categorical**						
0–1	1 (ref)		1 (ref)		1 (ref)	
2–3	1.164 (0.790, 1.715)	0.437	0.659 (0.470, 0.926)	0.017	0.685 (0.488, 0.960)	0.029
4–5	0.680 (0.452, 1.024)	0.064	0.638 (0.419, 0.972)	0.037	0.716 (0.469, 1.093)	0.119
≥6	0.172 (0.062, 0.477)	0.001	0.250 (0.089, 0.702)	0.009	0.313 (0.115, 0.854)	0.024
*P* for trend		0.02		< 0.001		0.004

OR, odds ratio; SDoH, social determinants of health.

All estimates were obtained from complex survey designs.

Model 1: adjusted for age and race/ethnicity.

Model 2: adjusted for age, race/ethnicity, body mass index, alcohol consumption, and smoking status.

To further assess the association between each SDoH factor and PCa prevalence, we adjusted for other SDoH using weighted multivariable logistic regression. The weighted mean prevalence rate, prevalence rate difference, and ORs are presented in Table 3. After adjusting for age, race/ethnicity, and other SDoH, a PIR < 3 (OR = 0.69, 95% CI: 0.499–0.954, *p* = 0.029) and lack of access to healthcare or reliance on emergency rooms (OR = 0.429, 95% CI: 0.218–0.842, *p* = 0.017) were independently negatively associated with PCa prevalence.

**Table 3:** Associations of each SDoH with PCa prevalence.

	**Prevalence (95% CI), %**	**Model 1**		**Model 2**	
		**OR (95% CI)**	***P*** **-value**	**OR (95% CI)**	***P*** **-value**
**Employment**			0.116		0.314
Employed, student, retired	3.39 (2.8, 3.97)	1 (ref)		1 (ref)	
Not employed	1.25 (0.59, 1.91)	0.653 (0.387, 1.102)		0.75 (0.431, 1.305)	
**Family income-to-poverty ratio**			0.003		0.029
≥3	2.86 (2.12, 3.6)	1 (ref)		1 (ref)	
< 3	3.33 (2.7, 3.96)	0.643 (0.487, 0.849)		0.69 (0.499, 0.954)	
**Food security**			0.604		0.793
Full food security	3.25 (2.59, 3.9)	1 (ref)		1 (ref)	
Marginal, low, or very low	1.78 (0.83, 2.73)	0.834 (0.421, 1.651)		1.109 (0.514, 2.39)	
**Education**			0.325		0.939
High school or more	2.87 (2.26, 3.49)	1 (ref)		1 (ref)	
Less than high school	3.85 (2.74, 4.96)	0.84 (0.596, 1.185)		0.986 (0.683, 1.422)	
**Access to healthcare**			0.008		0.017
Regular health-care facility	3.42 (2.8, 4.04)	1 (ref)		1 (ref)	
None or emergency room	0.52 (0.18, 0.86)	0.388 (0.197, 0.762)		0.429 (0.218, 0.842)	
**Health insurance**			0.254		0.632
Private insurance	2.94 (2.35, 3.54)	1 (ref)		1 (ref)	
Government or none	3.29 (2.27, 4.3)	0.79 (0.529, 1.18)		0.899 (0.583, 1.386)	
**Housing instability**			0.272		0.82
Own home	3.31 (2.66, 3.96)	1 (ref)		1 (ref)	
Rent or other arrangement	1.93 (1.14, 2.72)	0.777 (0.498, 1.213)		0.943 (0.571, 1.558)	
**Marital status**			0.235		0.497
Married or living with a partner	3.11 (2.48, 3.74)	1 (ref)		1 (ref)	
Not married nor living with a partner	2.86 (1.95, 3.78)	0.786 (0.53, 1.165)		0.864 (0.568, 1.313)	

All estimates were obtained from complex survey designs.

Model 1: adjusted for age and race/ethnicity.

Model 2: adjusted for age, race/ethnicity, and other SDoH.

### 3.3 Association of SDoH with highly-probable PCa

As shown in Supplementary Table S3, no significant association was found between continuous SDoH score and highly-probable PCa in the crude model (OR = 1.001, 95% CI: 0.950–1.055, *p* = 0.965), model 1 (OR = 0.989, 95% CI: 0.929–1.053, *p* = 0.728), and model 2 (OR = 0.987, 95% CI: 0.925–1.053, *p* = 0.68). Similarly, no significant association was found between categorical SDoH score and highly-probable PCa. In addition, we found no significant association between each SDoH factor and highly probable PCa (Supplementary Table S4).

### 3.4 Dose-response relationships

**Figure 2:**
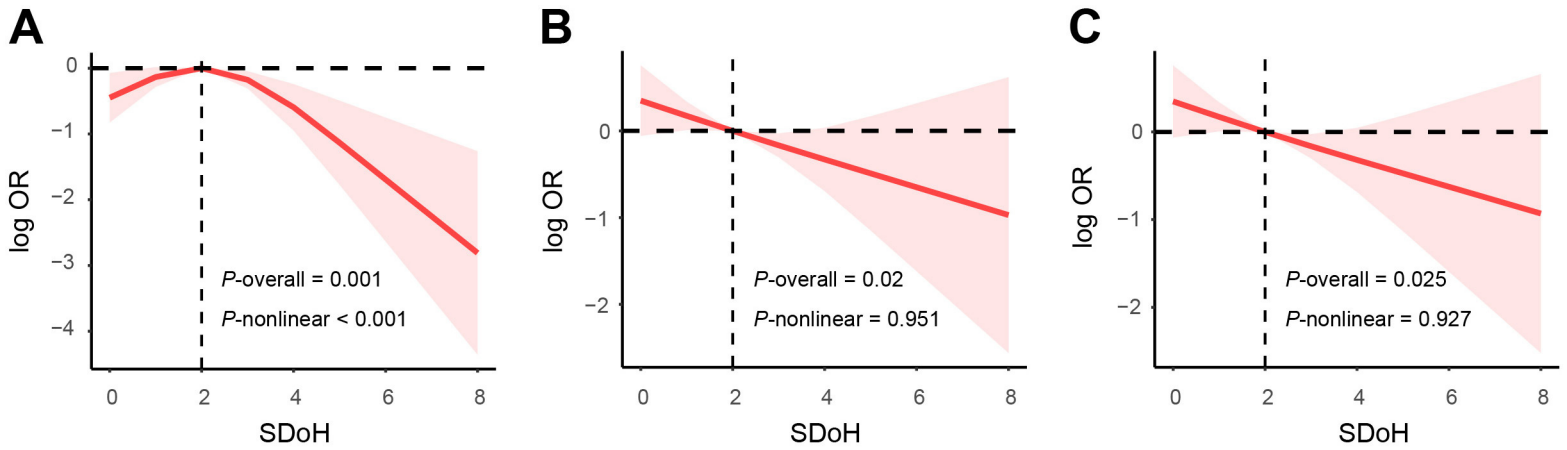
Dose-response relationship of SDoH with PCa prevalence (Optimal nKnots = 3). **(A)** crude model **(B)** adjusted for age and race/ethnicity **(C)** adjusted for age, race/ethnicity, body mass index, alcohol consumption, and smoking status.

Given the significant association between SDoH and PCa prevalence, we further investigated whether there is a dose-response relationship between them. In crude model (Figure 2A), the RCS analysis showed a nonlinear association between SDoH and PCa prevalence (*p*-overall = 0.001, *p*-non-linear < 0.001), with a cutoff value of 2. After adjusting for age and race/ethnicity (Figure 2B), a linear association was observed between SDoH and PCa prevalence (*p*-overall = 0.02, *p*-non-linear = 0.851). As shown in Figure 2C, this linear association remained after further adjustment for BMI, alcohol consumption, and smoking status (*p*-overall = 0.025, *p*-non-linear = 0.927).

### 3.5 Subgroup analysis

To determined whether the association between SDoH and PCa prevalence persists across specific populations, subgroups were stratified by age, race, and BMI (Supplementary Table S5). A consistently negative association was observed among individuals aged ≥ 60 years, Non-Hispanic Black, Non-Hispanic White, and non-obese (BMI < 30 kg/m^2^) groups. Interaction tests confirmed that the relationship between SDoH and PCa prevalence was not significantly influenced by age, race, or BMI.

## 4 Discussion

To our knowledge, this is the first study to investigate the associations between SDoH and the prevalence of PCa and highly probable PCa in a population-based setting. Despite statistically significant negative associations being observed between SDoH and PCa prevalence, no associations were identified between SDoH and the prevalence of highly probable PCa. After adjustment for potential confounding factors, the association between SDoH and PCa prevalence was found to be linear.

It is important to note that among unfavorable SDoH factors, a PIR < 3 and lack of access to healthcare or reliance on emergency rooms were identified as independently associated with lower PCa prevalence. A possible explanation for these findings is that individuals with low household income and limited healthcare access may have fewer opportunities for PCa diagnosis. Since PCa diagnosis was based on self-report from the questionnaire, where participants were asked if they had been told by a doctor or healthcare professional, some individuals with undiagnosed PCa may not have been aware of their condition. As a result, these individuals could have been misclassified as healthy in the survey due to a lack of access to screening. Epidemiological studies further support this explanation. For instance, across all US Surveillance, Epidemiology, and End Results registries, active surveillance was 20–31% more likely to be used for patients with median household incomes >$50,000 and $75,000, respectively (17). In addition, active surveillance was 28% less likely for eligible Medicaid patients compared to those with private insurance (17). Similarly, a PCa screening study in Switzerland found that men with higher socioeconomic status were more frequently screened than those with lower status (18).

Black men are twice as likely to develop the PCa and are more likely to be diagnosed with higher-grade PCa (3, 4, 19). Race is often a surrogate for socioeconomic status, and the literature suggests that both Black men and individuals with low socioeconomic status are less likely to undergo multiparametric magnetic resonance imaging (MRI) (13, 20). Our findings showed that unfavorable SDoH were negatively associated with PCa prevalence in both White and Black populations, suggesting that the issue of inadequate PCa screening may be widespread among individuals with unfavorable SDoH, rather than being limited to the Black population alone. In addition, PCa diagnoses and related mortalities are rare in men under 50 years of age, with ~85% of cases diagnosed after the age of 65. Our study found that unfavorable SDoH were associated with lower PCa prevalence in men over 60 years old, suggesting that some cases may go undiagnosed in this population due to lack of PCa screening. Furthermore, non-obese individuals may be more susceptible to the impact of SDoH on PCa screening, as this group tends to have higher positive detection rates under the same screening conditions. A cancer screening trial in the US found that individuals with higher BMI were less likely to screen positive via PSA tests or digital rectal exams and more likely to have inadequate screening (21). In addition, PSA testing varies significantly across racial and ethnic groups in the U.S., with the lowest screening rates observed among Hispanic, Asian, and Pacific Islander men in 2012 (22). In our study, these groups were classified under 'other race or ethnicity' due to their small proportions, a categorization consistent with the existing literature (7). Compared to Non-Hispanic White, these groups face more unfavorable SDoH and significant challenges in accessing healthcare and screening (7).

Notably, no association between SDoH and the prevalence of highly probable PCa was found, suggesting that unfavorable SDoH may not be a risk factor for the onset of PCa. In this study, highly probable PCa was defined based on PSA levels, with PSA density being the most commonly evaluated risk factor. However, a major challenge is the high rate of overdiagnosis when PSA is used as the primary marker for risk. Additionally, sociopolitical factors in the US contribute to overtreatment, with more than half of patients with low- and favorable intermediate-risk PCa undergoing unnecessary treatment (17). Recent results from a randomized controlled trial suggest that omitting biopsy in patients with negative MRI findings could eliminate more than half of the diagnoses of clinically insignificant PCa (23). However, individuals with unfavorable SDoH less likely to gets high-quality screening, diagnosis, and treatment, which may leading to poorer prognosis and premature death.

Healthy People 2030 prioritizes evidence-based cancer screening to reduce cancer deaths, with a specific goal for PCa prevention and control to reduce the death rate from the most recent 2022 data of 18.6 per 100,000 males to 16.9 per 100,000 males (24). However, disparities in cancer outcomes between populations of low and high socioeconomic status have persisted, and in some cases, widened over the past decades (9). This study clarifies the association between SDoH and PCa in the US population, providing actionable evidence to guide practice, research, and policy. While our findings suggest that unfavorable SDoH are associated with a lower prevalence of PCa, we interpret this as a result of inadequate screening, leading to underreporting, rather than as evidence that unfavorable SDoH protect against the disease. Therefore, we advocate for addressing SDoH to promote cancer health equity, particularly in the screening of PCa.

Our study has several limitations. First, as a cross-sectional analysis, it cannot establish a definitive causal relationship. Second, some important SDoH, such as family wealth, experiences of racism, discrimination, violence, and social support, were not available in NHANES. Third, because SDoH were only assessed at baseline, we were unable to evaluate the impact of changing SDoH over time. Fourth, residual confounding cannot be ruled out, such as dietary patterns, physical activity, and comorbidities, etc. Fifth, there is potential for misclassification in self-reported SDoH categories and PCa diagnoses. Sixth, the binary classification of SDoH used in this study may oversimplify the complexity of these factors. Specifically, the limited variables related to neighborhood and built environment, as well as social and community contexts, were considered. In our analysis, homeownership was used as a proxy for the neighborhood and built environment domain; however, other important factors such as neighborhood socioeconomic status and social support/perceived discrimination were not available in the NHANES dataset. Finally, while the recommendation for PSA screenings was updated in 2018, data on PCa and PSA testing are only available up to the 2009–2010 cycle, which may limit the generalizability of the findings to the present.

## 5 Conclusion

In conclusion, unfavorable SDoH are significantly associated with reduced PCa prevalence, with low income and lack of healthcare access independently linked to lower PCa prevalence. This is likely due to inadequate screening and underreporting rather than any protective effects. No significant associations were found between SDoH and highly probable PCa (defined by PSA levels), suggesting that unfavorable SDoH may not be a risk factor for the onset of PCa. These findings underscore the importance of addressing healthcare access disparities and improving cancer screening equity. Future research should focus on longitudinal assessments of SDoH and develop tailored interventions to promote health equity.

## Data Availability

Publicly available datasets were analyzed in this study. This data can be found here: https://www.cdc.gov/nchs/nhanes/.
