# Supplementary material for: Exploration of social determinants of health and prostate cancer prevalence in the US: a cross-sectional study of NHANES data from 2003 to 2010
Source: Front Public Health. 2025 Mar 12;13:1564498. doi: 10.3389/fpubh.2025.1564498 (PMC11936998; doi:10.3389/fpubh.2025.1564498)
Supplement: Supplementary file 1 [file Table_1.docx]

Supplementary Material

# Supplementary Tables

| **Table S1. Detailed descriptions of the SDoH-related questions and definitions** | | |
| --- | --- | --- |
| **SDoH variables** | **Category (score)** | **Questions and Definitions** |
| Employment status | Employed, student, or retired (0) | Participants were asked about their work activity in the past week and, if not working, the primary reason for not working. |
|  | Unemployed (1) |  |
| Family income-to-poverty ratio | ≥ 3 (0) | Participants were asked about their family income and size, and the poverty/income ratio was calculated as the ratio of family income to the poverty threshold. |
|  | < 3 (1) |  |
| Food security | Full security (0) | Participants responded to the following questions: 1) whether they were concerned food would run out before they had money to buy more; 2) if the food they bought didn’t last and they couldn’t afford more; 3) if they could not afford balanced meals; 4) if they had to cut meal sizes or skip meals due to lack of money for food; 5) if they had cut or skipped meals, how often this occurred; 6) if they ate less than they felt they should because of limited food funds; 7) if they were hungry but didn’t eat due to affordability issues; 8) if they lost weight due to insufficient money for food; 9) if they went without food for a full day because of financial constraints; 10) if they didn’t eat for a whole day, how often this occurred. Food security levels were classified: full food security (no affirmative responses), marginal food security (1-2 affirmative responses), low food security (3-5 affirmative responses), and very low food security (6-10 affirmative responses). |
|  | Marginal, low, or very low security (1) |  |
| Education level | High school graduate or above (0) | Participants were asked about the highest grade or level of school they completed. Response categories included: less than 9th grade education, 9-11th grade education (includes 12th grade and no diploma), High school graduate/GED, some college or associates (AA) degree, and college graduate or higher. |
|  | Less than high school (1) |  |
| Regular health-care access | At least one regular health-care facility (0) | Participants were asked if they have a usual place they go when sick or need health advice.Those who answered "yes" or "there is more than one place" were classified as having a routine healthcare. |
|  | None or emergency room (1) |  |
| Type of health insurance | Private (0) | Participants were asked if they have health insurance or another type of healthcare plan.They were then asked to specify whether they are covered by private insurance or by various types of government insurance, including Medicare, Medi-Gap, Medicaid, SCHIP, military healthcare, Indian Health Service, state-sponsored health plans, or other government insurance. |
|  | Government or none (1) |  |
| Home ownership | Own home (0) | Participants were asked whether the home they live in is owned, being bought, rented, or occupied through another arrangement. A person was considered to own the home even if they are still paying off a mortgage. |
|  | Rent home or other arrangement (1) |  |
| Marital status | Married or living with a partner (0) | Participants were asked about their marital status, with options including married, widowed, divorced, separated, never married, or living with a partner. |
|  | Not married nor living with a partner (1) |  |

| **Table S2. Characteristics of study participants who underwent PSA testing** | | | | |
| --- | --- | --- | --- | --- |
| **Variable** | **Total**  **(n = 5,072)** | **Normal**  **(n = 3,860)** | **Highly-probable PCa (n = 1,212)** | **P-value** |
| Age (years) | 53(46,63) | 52(46,60) | 64(55,73) | < 0.0001 |
| Race/ethnicity (%) |  |  |  | 0.1 |
| Mexican American | 884( 6.1) | 715(6.4) | 169(4.6) |  |
| Non-Hispanic Black | 904( 8.6) | 671(8.4) | 233(9.6) |  |
| Non-Hispanic White | 2810(78.0) | 2107(77.8) | 703(79.1) |  |
| Other | 474( 7.3) | 367(7.4) | 107(6.7) |  |
| BMI category (%) |  |  |  | 0.02 |
| Underweight | 53( 0.8) | 32(0.7) | 21(1.1) |  |
| Normal | 1132(20.8) | 831(20.5) | 301(22.1) |  |
| Overweight | 2107(42.2) | 1591(41.6) | 516(45.1) |  |
| Obese | 1780(36.3) | 1406(37.3) | 374(31.7) |  |
| Alcohol consumption (%) |  |  |  | < 0.001 |
| Former | 1326(21.2) | 954(20.4) | 372(24.4) |  |
| Never | 324( 5.6) | 221(5.0) | 103(8.0) |  |
| Now | 3422(73.3) | 2685(74.5) | 737(67.7) |  |
| Smoking status (%) |  |  |  | < 0.001 |
| Former | 2004(36.8) | 1448(35.3) | 556(43.2) |  |
| Never | 1924(41.4) | 1480(41.9) | 444(38.9) |  |
| Now | 1144(21.9) | 932(22.8) | 212(17.9) |  |
| Employment (%) |  |  |  | 0.05 |
| Employed, student, retired | 4143(84.2) | 3114(83.6) | 1029(86.8) |  |
| Not employed | 929(15.8) | 746(16.4) | 183(13.2) |  |
| Family income-to-poverty ratio (%) |  |  |  | 0.1 |
| ≥ 3 | 2177(59.7) | 1690(60.6) | 487(55.8) |  |
| < 3 | 2895(40.3) | 2170(39.4) | 725(44.2) |  |
| Food security (%) |  |  |  | 0.04 |
| Full food security | 4047(86.8) | 3030(86.3) | 1017(89.2) |  |
| Marginal, low, or very low | 1025(13.2) | 830(13.7) | 195(10.8) |  |
| Education (%) |  |  |  | 0.03 |
| High school or more | 3537(82.0) | 2723(82.6) | 814(79.5) |  |
| Less than high school | 1535(18.0) | 1137(17.4) | 398(20.5) |  |
| Access to healthcare (%) |  |  |  | 0.002 |
| Regular health-care facility | 4342(86.7) | 3239(85.7) | 1103(91.1) |  |
| None or emergency room | 730(13.3) | 621(14.3) | 109( 8.9) |  |
| Health insurance (%) |  |  |  | 0.02 |
| Private insurance | 2881(68.7) | 2235(69.7) | 646(64.0) |  |
| Government or none | 2191(31.3) | 1625(30.3) | 566(36.0) |  |
| Housing instability (%) |  |  |  | 0.3 |
| Own home | 3819(81.4) | 2876(81.0) | 943(83.0) |  |
| Rent or other arrangement | 1253(18.6) | 984(19.0) | 269(17.0) |  |
| Marital status (%) |  |  |  | 0.8 |
| Married or living with a partner | 3710(77.0) | 2848(77.1) | 862(76.7) |  |
| Not married nor living with a partner | 1362(23.0) | 1012(22.9) | 350(23.3) |  |
| SDoH score (%) |  |  |  | 0.3 |
| 0-1 | 2061(56.1) | 1584(56.5) | 477(54.3) |  |
| 2-3 | 1587(26.2) | 1168(25.5) | 419(29.1) |  |
| 4-5 | 1050(13.5) | 810(13.6) | 240(12.8) |  |
| ≥ 6 | 374( 4.2) | 298(4.3) | 76(3.7) |  |
| Total prostate-specific antigen (ng/mL) | 0.88(0.54,1.60) | 0.73(0.50,1.10) | 3.07(2.43,4.80) | < 0.0001 |
| Free prostate-specific antigen (ng/mL) | 0.27(0.17,0.42) | 0.23(0.16,0.33) | 0.68(0.49,1.01) | < 0.0001 |
| The data are presented as the mean (95% CI) or number (%). All estimates were obtained from complex survey designs, using analysis of variance or chi-square tests as appropriate. | | | | |

| **Table S3. Association of SDoH score with highly-probable PCa** | | | | | | |
| --- | --- | --- | --- | --- | --- | --- |
| **SDoH score** | **Crude model** | | **Model 1** | | **Model 2** | |
|  | **OR (95%CI)** | ***P*** | **OR (95%CI)** | ***P*** | **OR (95%CI)** | ***P*** |
| **Continuous** | 1.001(0.950,1.055) | 0.965 | 0.989(0.929,1.053) | 0.728 | 0.987(0.925,1.053) | 0.680 |
| **Categorical** |  |  |  |  |  |  |
| 0-1 | 1 (ref) |  | 1 (ref) |  | 1 (ref) |  |
| 2-3 | 1.183(0.929,1.508) | 0.169 | 0.871(0.684,1.110) | 0.259 | 0.875(0.685,1.116) | 0.275 |
| 4-5 | 0.979(0.747,1.283) | 0.875 | 1.004(0.740,1.361) | 0.982 | 0.993(0.726,1.359) | 0.965 |
| 6+ | 0.899(0.640,1.262) | 0.531 | 1.116(0.779,1.598) | 0.543 | 1.092(0.756,1.577) | 0.632 |
| *P* for trend |  | 0.963 |  | 0.933 |  | 0.871 |
| Model 1: adjusted for age and race/ethnicity.  Model 2: adjusted for age, race/ethnicity, body mass index, alcohol consumption, and smoking status. | | | | | | |

| **Table S4. Associations of each SDoH with highly-probable PCa** | | | | |
| --- | --- | --- | --- | --- |
|  | **Model 1** | | **Model 2** | |
|  | **OR (95%CI)** | ***P*-value** | **OR (95%CI)** | ***P*-value** |
| **Employment** |  | 0.893 |  | 0.976 |
| Employed, student, retired | 1 (ref) |  | 1 (ref) |  |
| Not employed | 0.983 (0.763, 1.265) |  | 0.996 (0.764, 1.298) |  |
| **Family income-to-poverty ratio** |  | 0.27 |  | 0.168 |
| ≥ 3 | 1 (ref) |  | 1 (ref) |  |
| < 3 | 0.876 (0.694, 1.106) |  | 0.844 (0.665, 1.071) |  |
| **Food security** |  | 0.903 |  | 0.944 |
| Full food security | 1 (ref) |  | 1 (ref) |  |
| Marginal, low, or very low | 0.983 (0.747, 1.294) |  | 1.01 (0.763, 1.338) |  |
| **Education** |  | 0.207 |  | 0.356 |
| High school or more | 1 (ref) |  | 1 (ref) |  |
| Less than high school | 0.888 (0.739, 1.066) |  | 0.904 (0.73, 1.118) |  |
| **Access to healthcare** |  | 0.957 |  | 0.918 |
| Regular health-care facility | 1 (ref) |  | 1 (ref) |  |
| None or emergency room | 1.01 (0.715, 1.426) |  | 1.019 (0.72, 1.441) |  |
| **Health insurance** |  | 0.728 |  | 0.442 |
| Private insurance | 1 (ref) |  | 1 (ref) |  |
| Government or none | 1.04 (0.836, 1.293) |  | 1.094 (0.872, 1.372) |  |
| **Housing instability** |  | 0.408 |  | 0.214 |
| Own home | 1 (ref) |  | 1 (ref) |  |
| Rent or other arrangement | 1.121 (0.857, 1.467) |  | 1.174 (0.915, 1.506) |  |
| **Marital status** |  | 0.906 |  | 0.712 |
| Married or living with a partner | 1 (ref) |  | 1 (ref) |  |
| Not married nor living with a partner | 0.988 (0.811, 1.204) |  | 0.967 (0.81, 1.154) |  |
| All estimates were obtained from complex survey designs.  Model 1: adjusted for age and race/ethnicity.  Model 2: adjusted for age, race/ethnicity, and other SDoH. | | | | |

| **Table S5.** Subgroup analyses of the association between SDoH and PCa prevalence | | | |
| --- | --- | --- | --- |
| **Subgroups** | **OR (95% CI)** | ***P*** | ***P* for interaction** |
| **Age group** |  |  | 0.125 |
| 40-59 | 0.707 (0.474, 1.055) | 0.088 |  |
| ≥ 60 | 0.891 (0.803, 0.988) | 0.029 |  |
| **Race/ethnicity** |  |  | 0.682 |
| Non-Hispanic White | 0.859 (0.750, 0.984) | 0.030 |  |
| Non-Hispanic Black | 0.832 (0.730, 0.948) | 0.007 |  |
| Mexican American | 0.956 (0.708, 1.291) | 0.763 |  |
| Other | 0.698 (0.440, 1.106) | 0.123 |  |
| **BMI category** |  |  | 0.168 |
| Underweight | 0.494 (0.249, 0.982) | 0.046 |  |
| Normal | 0.786 (0.629, 0.983) | 0.035 |  |
| Overweight | 0.818 (0.672, 0.995) | 0.044 |  |
| Obese | 0.966 (0.864, 1.080) | 0.538 |  |
| OR, odds ratio; BMI, body mass index.  Adjusted for age, race/ethnicity, BMI, alcohol consumption, and smoking status. | | | |
